# Spanish Pediatricians’ Positions Regarding Prevention, Diagnosis, Nutritional Management, and Challenges in Cow’s Milk Protein Allergy

**DOI:** 10.3390/nu15163586

**Published:** 2023-08-15

**Authors:** Rafael Martín-Masot, Juan José Díaz-Martín, Alicia Santamaría-Orleans, Víctor Manuel Navas-López

**Affiliations:** 1 Pediatric Gastroenterology and Nutrition Unit, Hospital Regional Universitario de Málaga, 29011 Málaga, Spainvictor.navas@gmail.com (V.M.N.-L.); 2 Pediatric Gastroenterology and Nutrition Unit, Hospital Universitario Central de Asturias, 33011 Oviedo, Spain; 3 Laboratorios Ordesa S.L., Scientific Communication Department, 08830 Sant Boi del Llobregat, Spain; orleans1970@gmail.com

**Keywords:** cow’s milk protein allergy, diagnosis, prevention, treatment, extensively hydrolyzed formula, hydrolyzed rice protein-based formulas, amino acid formulas

## Abstract

Management of cow’s milk protein allergy (CMPA) can vary depending on the experience and area of expertise of the clinician responsible for the patient’s follow-up, which may or may not align with the recently published literature. To analyze the perspectives of Spanish pediatricians on this topic, a survey was conducted. The survey aimed to determine the current opinions and attitudes of 222 primary care and hospital pediatricians toward CMPA prevention and nutritional management. Participating pediatricians completed the questionnaire, providing insights into their daily clinical practices, including access to testing, attitudes with respect to various aspects of CMPA diagnosis, prevention, oral food challenges, and treatment. The findings revealed that pediatricians generally agree on the use of extensively hydrolyzed formulas (eHFs) to prevent CMPA in high-risk atopic children, despite limited evidence supporting the widespread use of this practice. However, consensus was lacking regarding the utility of formulas with prebiotics and probiotics for expediting tolerance development. In most cases, pediatricians preferred eHFs for the nutritional management of CMPA, followed by hydrolyzed rice formulas (HRFs), with amino-acid-based formulas (AAFs) being the third option. Certain issues remained controversial among pediatricians, such as prevention methods, symptom assessment, and the role of probiotics. These variations in management approaches reflect the influence of clinician experience and area of expertise, underscoring the need for standardized guidelines in this field.

## 1. Introduction

Cow’s milk protein allergy (CMPA) is the most common allergy observed among infants and young children [1]. The immune reaction to cow’s milk protein (CMP) can manifest as IgE-mediated, non-IgE-mediated, or mixed allergies, leading to variations in clinical presentation and symptom severity [2]. According to the EuroPrevall study [3], 0.54% of European children under the age of 2 develop CMPA. In the Spanish population, the incidence of IgE-mediated CMPA during the first year of life is reported to be 0.36% [4]. Unlike allergies to other foods, CMPA often resolves spontaneously, with most children developing a tolerance before 3 years of age, particularly in non-IgE-mediated cases. However, despite its prevalence, the management of CMPA varies significantly based on the experience and area of expertise of the clinician responsible for the patient’s follow-up [5].

Various medical and scientific organizations have published a series of guidelines in high-impact journals to standardize the management of CMPA among pediatric specialists, including primary care pediatricians, pediatric gastroenterologists, and pediatric allergists [6], like the ones recently published by the ESPGHAN in July 2023 [7]. While the indications for diagnosis and nutritional management are well-defined, there remains ongoing controversy and discussion regarding certain aspects of CMPA management, such as prevention methods, symptom assessment, and the promotion of tolerance development.

An example that highlights the evolving perspectives is the occasional supplementation of breastfed infants with infant formulas during the first days of life and the potential protective role displayed when an extensively hydrolyzed formula is used. Between 1999 and 2014, several societies recommended such supplementation for children at risk of atopic disease [8,9,10]. However, in 2016, a new recommendation emerged, advocating for the early introduction of cow’s-milk-based infant formulas when supplementing breastfeeding, regardless of the child’s atopic risk. This shift was based on large observational studies suggesting a reduced incidence of food allergies, including milk, eggs, and peanuts, through early exposure to potentially allergenic foods [11,12,13]. In our setting, a recent consensus document focused on the prevention of CMPA in infants under 7 days of age concluded that the current evidence does not support making a general recommendation to avoid cow’s milk protein in the first week of life. However, there is some evidence suggesting the beneficial role of avoidance in children at a higher risk of atopy [6]. These findings highlight the ongoing complexity and evolving understanding of CMPA prevention strategies in the early stages of life.

Similarly, there is evidence of bovine protein fragments being transmitted through breast milk; it has been suggested that this transmission may play a role in initiating a sensitization process to CMP in children with a high risk of developing atopic conditions [14]. Recent systematic reviews, including the 2019 report from the American Academy of Pediatrics [15], have concluded that there is insufficient evidence to support dietary restrictions for mothers during pregnancy and lactation. These findings align with the latest recommendations, emphasizing the relevance of early exposure to CMP for generating both early sensitization and tolerance [15].

Another controversial issue regarding the progression of CMPA is the influence of gastrointestinal microbiota, including the diversity and intensity of bacterial exposure, on the development of tolerance to CMP [16,17]. While some studies suggest potential benefits, there is currently insufficient evidence to support the use of probiotics, prebiotics, symbiotics, fecal microbiota transplant, or other microbiota-modulating strategies for the treatment of CMPA. As a result, these approaches have not been directly incorporated into national or international guidelines [18]. The existing evidence only applies to specific strains of probiotics and cannot be extrapolated to all probiotic strains, genera, concentrations, or administration timings. Therefore, until new clinical evidence becomes available, these approaches are not likely to be widely adopted in clinical practice or officially recommended [19]. When analyzing clinical practice related to CMPA, several years ago, the Portuguese Association of Pediatric Allergology (SPAP) and the Spanish Society of Pediatric Allergology and Clinical Immunology (SEICAP) published a consensus statement. This consensus, developed during the 2014–2015 period, addressed various aspects of CMPA management, including prevention, diagnosis, treatment, and progression [5]. However, considering that this consensus represented the perspective of allergy specialists, and a considerable amount of time has passed since its publication, it is worth examining whether the opinions and knowledge of non-specialized pediatricians differ from those expressed in the previous study due to differences in their work environments and varying access to clinical examinations. To address this topic, a multicenter, cross-sectional descriptive study was conducted. Its objective was to assess the current opinions and positions of primary care and hospital pediatricians regarding CMPA, as well as their personal experiences in managing CMPA in their respective medical consultations. Additionally, this study aimed to compare these findings with the previously recorded views of other specialists [5].

## 2. Materials and Methods

A systematic search was conducted by two independent reviewers in the following databases: PubMed, Embase, and the Cochrane Central Register of Controlled Trials (CENTRAL). The search terms used in PubMed included “cow’s milk protein allergy” and “consensus”. The titles and abstracts of the identified articles were examined and the selection of articles was agreed upon by the two investigators. Selected articles were then thoroughly reviewed in their full text and, based on them, the final questionnaire for the project was constructed (see Supplementary Materials). The questionnaire considered the questions used in the Iberian consensus on cow’s milk allergy [5], which involved pediatric allergists from Spain and Portugal. These questions were used, in part, to compare the experiences and positions of the pediatricians participating in this study. During the second half of 2021, a large sample of primary care (PC) and hospital pediatricians with experience in managing children with CMPA from various regions across Spain were invited to participate. They were asked to complete a comprehensive questionnaire that covered various aspects of their daily clinical practice in managing CMPA (Supplementary Materials File S1). The questionnaire included topics such as their work setting; options for accessing and performing different tests based on the child’s CMPA profile; and their perspectives on diagnosis (7 items), prevention (6 items), challenge tests (8 items), and nutritional management (15 items) involving the use of milk protein hydrolysates, other plant-based proteins, or elemental formulas.

Responses regarding the level of agreement with the questionnaire items were recorded on a 5-point scale, including “completely disagree”, “disagree”, “neutral”, and “agree”, and “completely agree”. For the sake of simplicity, response categories were grouped into “disagree”, “neutral”, and “agree” and these groupings are presented in tables.

Categorical variables were reported as frequencies and percentages. Variables with a normal distribution were expressed as mean ± standard deviation and those without a normal distribution were expressed as median and interquartile range (IQR). We employed the Kolmogorov–Smirnov test to evaluate the normality of the distribution. A comparison between the variables was conducted using Fisher’s exact test for categorical variables, the Student’s *t*-test, or the ANOVA test for continuous variables, depending on whether the comparison involved two or more groups, respectively. All statistical analyses were performed using the SAS statistical package version 9.4, with a statistical significance value set at 0.05.

## 3. Results

A total of 222 pediatricians participated in the project, whose demographic and occupational characteristics are described in Table 1.

Regarding the management of CMPA at primary care centers (PCCs), and in the presence of symptoms suggestive of an IgE-mediated allergy, 41% of the pediatricians request a measurement of CMP-specific IgE in blood and 54.95% also request a skin prick test (SPT) for diagnosis. In terms of the ability to perform these types of measurements, the overall percentage was 54.5% among the surveyed pediatricians, with statistically significant differences between the pediatricians in hospitals (75%) and PCCs (24.9%) (*p* < 0.001). Concerning the ability to perform an oral food challenge (OFC) for CMPA, it is noteworthy that 54.84% of pediatricians from PCCs mentioned that these tests are not performed in their workplaces. However, they are generally performed in hospital centers, with the responsible departments being Pediatric Allergology (47.3%), Pediatric Gastroenterology (28.83%), and Pediatrics (22.97%).

In terms of the nutritional management of patients under 6 months of age with IgE-mediated CMPA, extensively hydrolyzed formulas (eHFs) are the preferred choice in 90.99% of cases, followed by hydrolyzed rice protein formulas (HRFs) (13.51%) and amino-acid-based formulas (AAFs) (5.41%). This trend is observed for both an IgE-mediated allergy and non-mediated allergy, regardless of the infant’s age (Table 2).

It is important to note that soy-based formulas (SFs) are used in very few cases, accounting for only up to 5.86% of patients over 6 months of age with a non-IgE-mediated allergy. No significant differences were found depending on the work setting.

The opinions and perceptions of pediatricians regarding various aspects of diagnosis, prevention, challenge tests, and nutritional management are presented in Table 3, Table 4, Table 5 and Table 6. These tables display the cumulative percentages of disagreement, neutral positioning, and agreement with the statements, as well as the mean values and standard deviations of the response scores (ranging from one to five). These values allow for a direct comparison of opinions based on the healthcare setting, whether at a PCC or hospital.

## 4. Discussion

### 4.1. Prevention Domain

There is ongoing debate surrounding the effectiveness of various preventive measures in reducing the risk of allergy development, particularly food allergies, such as CMPA. While it is evident that breastfeeding until at least 6 months of age may contribute to reducing the incidence of atopic dermatitis [5], its role in preventing CMPA and other food allergies is limited [20]. Notably, the imposition of dietary restrictions beyond those necessitated for maternal well-being during pregnancy and lactation is not warranted to forestall CMPA occurrence [7].

The conjecture surrounding the avoidance or delayed introduction of cow’s milk-based formulas to modulate the incidence of CMPA in high-risk infants lacks substantive scientific corroboration. The precise role of such interventions in altering the intricate immune dynamics underpinning CMPA predisposition remains enigmatic [7].

Regarding the potential mitigation of CMPA through abstention from the regular consumption of cow’s-milk-based formulas during early infancy, the linkage between this practice and a concrete reduction in subsequent CMPA risk in childhood remains elusive. Furthermore, the commencement of supplementary feeding, encompassing formulations other than breast milk during the nascent days of an infant’s life, is contraindicated in the context of CMPA prevention.

In the scenario of infants harboring a documented familial predisposition to allergic disorders, for whom exclusive breastfeeding is not viable, the routine adoption of partially hydrolyzed formula (pHF), extensively hydrolyzed formula with whey (eHF-Whey), or extensively hydrolyzed formula with casein (eHF-Casein), as CMPA preventive measures, lacks substantive empirical underpinning [21]. Moreover, the prospective role of extensively hydrolyzed formula (HRF) in the intricate tapestry of CMPA prevention remains a domain largely unexplored [7,22,23]. Similarly, the utilization of soy formula as a prophylactic measure against CMPA in infants with a verified ancestral allergic disease lineage who cannot be solely breastfed is bereft of compelling evidential support.

In any case, these trends previously mentioned in different publications have influenced pediatric health professionals as 60.4% of the surveyed professionals agreed on the preventive effect of extensively or partially hydrolyzed formulas in the development of atopic diseases compared to formulas with intact milk proteins. Additionally, 59.5% believed that administering eHF to high-risk infants from birth to six months can help prevent CMPA. Moreover, 80% of the surveyed pediatricians agreed that formulas enriched with prebiotics or probiotics have some preventive benefits for atopic dermatitis; although, there is insufficient evidence to recommend their routine use [7]. Hospital pediatricians were more likely to hold this opinion compared to primary care physicians (4.1 ± 0.92 vs. 3.7 ± 1; *p* < 0.005).

Another controversial topic discussed earlier is the exposure to cow’s milk during the first week of life in infants receiving mixed feeding; this is due to the potential risks of allergy. While a 2021 publication by the European Society of Allergology and Clinical Immunology (EAACI) recommended using an extensively hydrolyzed formula together with breastfeeding during the first week to prevent sensitization to milk proteins [24], a consensus document among various Spanish pediatric societies concluded that, taking into account the current level of evidence, a clear guideline cannot be established, despite certain studies showing some benefit in avoiding cow milk consumption during the first week of life [6].

Along the same lines of early allergen exposure, particularly through breast milk, more than half of the pediatricians (56.4%) recommended not excluding cow’s milk and its derivatives from the mother’s diet to prevent sensitization in high-risk infants, based on published evidence [7,25]. This recommendation was more commonly accepted by hospital pediatricians (2.0 ± 1.22 vs. 3 ± 1.47; *p* < 0.05).

Overall, the responses from the pediatricians in this study were largely consistent with those of the pediatric allergists in the CIBAL study [5] when considering these specific questions.

### 4.2. Diagnosis Domain

The diagnosis of CMPA relies on clinical manifestations, diagnostic tests, and responses to the challenge test, as outlined in consensus statements and clinical guidelines [26,27]. There is a high level of agreement that the rapid onset of symptoms (within two hours) following the consumption of infant formula or dairy foods strongly suggests IgE-mediated CMPA (94.6%). Pediatricians also recognize that the systematic rejection of the feeding bottle, accompanied by crying and irritability, without other signs of disease, can be symptomatic of CMPA (63.9%). Primary care pediatricians demonstrated greater agreement on this statement compared to hospital ones (4.1 ± 0.79 vs. 3.6 ± 0.93, *p* < 0.005). Moreover, the disappearance of symptoms or significant clinical improvement after switching to an extensively hydrolyzed formula is considered supportive of a CMPA diagnosis (78.8%). Primary care pediatricians showed greater agreement on this statement as well (3.9 ± 0.98 vs. 3 ± 1.4; *p* < 0.0001).

In terms of the role of specific IgEs in the diagnosis, there is disagreement among pediatricians. While it is considered that specific IgEs indicate sensitization rather than the presence of an allergy and higher values may suggest a longer time required for tolerance acquisition, nearly half of the pediatricians disagreed with the notion that the diagnosis of CMPA can be primarily based on positive results in tests measuring allergen-specific IgE. On the other hand, six out of ten pediatricians considered the SPT with cow’s milk and specific milk proteins (alpha-lactalbumin, beta-lactoglobulin, and casein) a valid method for diagnosing CMPA in infants at any age. Hospital pediatricians demonstrated higher agreement on this statement compared to primary care pediatricians (3.8 ± 1.1 vs. 3.2 ± 1.2; *p* < 0.005); although, the agreement was lower compared to the 94.41% agreement reported by pediatric allergists in other surveys [5].

### 4.3. Nutritional Management Domain

The nutritional management of CMPA involves the exclusion of cow’s milk protein from the diet; nearly all pediatricians (96.4%) agreed on the relevance of strictly removing milk proteins from the diet and educating patients about foods that may contain them or cross-reactive proteins.

If breastfeeding is not possible, substitution formulas should be included in the infant’s diet. Most guidelines recommend eHF as the first-choice formula, followed by AAF in severe cases; in some instances, HRF and SF are considered viable options [7,28]. A total of 94.6% of the surveyed pediatricians agreed with this statement. 

Previous concerns about the use of HRF regarding the protein quality adequacy (lysine deficiency), rice allergies, and arsenic content have been refuted by recent studies, which demonstrate the safety of these formulas in children with CMPA and their ability to support normal growth [29,30,31].

This reassurance regarding HRFs is reflected in our survey, where they were indicated as the second preferred alternative after eHFs and before EFs. The increased use of HRFs compared to previous studies confirms the confidence of pediatricians in their safety and effectiveness in promoting adequate growth in the children who consume them. The use of soy protein formulas was observed in less than 5% of children under and over 6 months of age.

However, when it was directly asked, 78.4% of the pediatricians agreed that soy protein formulas are not nutritionally adequate for infants under 6 months of age due to their phytoestrogen content and/or the risk of soy sensitization. While the perception of soy sensitization in infants with CMPA may be more prevalent in English-speaking countries, Spanish studies have shown a low rate (4%) of CMPAs with sensitization accompanied by clinical expression, which calls into question the necessity of soy protein formulas [32]. Regarding taste preferences, more than 80% of pediatricians believed that HRFs are better accepted by infants in terms of organoleptic qualities compared to eHFs. However, palatability is a subjective factor reported by adults and other studies with pediatricians contradict the belief that eHFs have poor taste and are rejected by infants [5]. Regarding the elimination of cow’s milk from the diet, even if the patient tolerates it despite sensitization (positive SPT or specific IgE test), pediatricians generally agreed (72.3%) that it should not be applied due to the risk of losing tolerance (65%) and triggering severe allergic reactions upon reintroduction [33].

Hospital pediatricians showed stronger agreement on this statement compared to the primary care pediatricians (4.3 ± 1.02 vs. 3.6 ± 1.27; *p* < 0.001); but, the level of agreement was lower than that reported in the CIBAL study, where over 90% of pediatric allergists agreed with this position [5]. There was a broad consensus (86.5%) that 80% of infants will develop tolerance to cow’s milk by the age of 2. However, there was disagreement (58.5% disagree) regarding the statement that IgE-mediated allergies will develop tolerance earlier than non-IgE-mediated allergies. The role of specific probiotics in promoting the accelerated development of tolerance in infants with CMPA remains unclear, despite the existence of numerous publications suggesting that formulas supplemented with symbiotics, various prebiotics, and probiotics generally lead to a reduction in symptoms, particularly those related to allergies [34,35]. It is worth noting that opinions in the field of nutritional management, including those concerning this aspect, were consistent with those documented by pediatric allergists in the CIBAL study [5].

### 4.4. Challenge Test Domain

An oral food challenge (OFC) is essential in assessing CMPA in infants, except for life-threatening anaphylaxis cases and instances of high sIgE levels. However, parents and healthcare providers often decline OFCs. OFCs should begin with tiny milk doses, adjusted for severe immediate reactions, sometimes starting with lip application followed by incremental dosing. If no reaction occurs, the at-home continuation of milk administration is advised. Hospital OFCs are preferred in cases of a history of immediate reactions, unpredictability, or severe eczema. Intravenous access is reserved for severe reactions. In mild–moderate non-IgE CMPA cases, milk introduction might be performed at home under adequate healthcare supervision. In severe non-IgE cases (i.e., FPIES), or in those cases where there are doubts about the underlying mechanism (reaction in the first two hours after milk ingestion, positive specific IgE to cow’s milk proteins), the home challenge is not recommended [2]. The double-blind placebo-controlled food challenge (DBPCFC) is the diagnostic gold standard but is resource-intensive, limiting its use in clinical practice [7].

The majority of pediatricians (60.9%) concluded that a challenge test is essential for the diagnosis of CMPA and agreed that it should be performed in a hospital setting (61.5% agreed and 26.1% disagreed). This position is possibly influenced by the healthcare setting in which the surveyed pediatricians work as most of them (61.25%) practice in hospitals and have extensive experience in clinical situations.

A significant proportion (92.7%) considered CMPA to be in clinical remission and would allow cow’s milk inclusion in the diet if the controlled OFC yielded negative results. They recommend the regular consumption of cow’s milk at home for two weeks when there is good tolerance, even if IgE sensitization persists and is indicated by a positive SPT or high serum IgE levels. If the challenge test is positive, 82.2% of the surveyed pediatricians agreed that it should not be repeated within the next six months and 57.8% stated that it should not be repeated before the child reaches 12 months of age. This viewpoint had greater support among primary care pediatricians (3.9 ± 1.24 vs. 3.1 ± 1.42; *p* < 0.001).

In contrast to the allergists in the CIBAL study (96.6%), only 52.1% of the pediatricians considered it unnecessary to wait for negative results in the SPT and serum IgE test before assessing tolerance using a controlled challenge test. Finally, 88.6% of the respondents considered a cautious and controlled challenge test for milk as the only definitive tool to assess whether there has been progress toward tolerance.

## 5. Conclusions

This study presents the current opinions regarding nutritional management and approaches to CMPA among a large group of Spanish pediatricians in both primary care and hospital settings. The findings indicate that the opinions recorded in this study are generally similar across different work settings and align closely with those of the pediatric allergists in a previous study.

In terms of CMPA prevention, there is consensus among pediatricians regarding the use of eHF in high-risk atopic patients, despite the limited scientific evidence supporting the widespread adoption of this practice. However, there is no consensus on the effectiveness of formulas supplemented with prebiotics and probiotics to accelerate tolerance development.

The preferred formulas for the nutritional management of CMPA are eHFs followed by HRPFs; AAFs are considered the third option. The use of SFs by Spanish pediatricians is minimal.

While the recorded opinions between primary care and hospital pediatricians did not show significant differences for the majority of the analyzed items, the lack of consensus on certain points is noteworthy; this should be considered particularly relevant because the variability in clinical practice can be a determining factor in the delivery of low-quality care.

The publication of standardized guidelines that encompass strategies for CMPA prevention, diagnostic approaches, and nutritional management is needed to offer actionable insights to guide healthcare professionals when dealing with cases of CMPA.

## Figures and Tables

**Table 1:** Profile of participating pediatricians. Data are expressed as percentages and means (standard deviation).

**Participating Pediatricians (n)**	222	
**Age (median, SD)**	51.46 (12.05) years	
	**≤50 years**	**>50 years**
	40.09%	58.11%
**Sex**	**Male**	**Female**
	60.81%	39.19%
**Care Setting**	**Primary Care**	**Hospital**
	38.75%	61.25%
**Years of experience (median, SD)**	25.29 (10.48)	

SD: standard deviation.

**Table 2:** Type of infant formulas recommended based on infant’s age (<6 and ≥6 months) and type of allergy (IgE-mediated/Non-IgE-mediated).

	<6 Months				≥6 Months			
	IgE-Mediated		Non-IgE-Mediated		IgE-Mediated		Non-IgE-Mediated	
	N	%	N	%	N	%	N	%
Extensively hydrolyzed formulas	202	90.99	196	88.29	197	88.74	177	79.73
Hydrolyzed rice protein formulas	30	13.51	51	22.97	51	22.97	56	25.23
Elemental formulas	12	5.41	6	2.7	10	4.5	6	2.7
Soy protein formulas	2	0.9	0	0	3	1.35	13	5.86
Other	7	3.15	1	0.45	2	0.9	3	1.35

**Table 3:** Results of the “Prevention” domain. Answers range from one (completely disagree) to five (completely agree). Means and SDs for each category. Overall data, categorized by work setting and significance for comparison * *p* < 0.05.

Prevention	Overall					Primary Care	Hospital	
Variable	N	Disagree (%)	Neutral (%)	Agree (%)	Mean (SD)	Mean (SD)	Mean (SD)	
Partially hydrolyzed and extensively hydrolyzed formulas have a preventive effect for atopic disease compared to formulas containing intact proteins	220	23.60	15.9	60.4	3.5 (1.28)	3.5 (1.26)	3.3 (1.25)	-
Extensively hydrolyzed formulas given to infants at high atopic risk from birth to 6 months to supplement or replace breast milk are more effective than partially hydrolyzed formulas to prevent CMPA	220	17.70	22.7	59.5	3.6 (1.23)	3.9 (1.13)	3.3 (1.28)	*
Formulas enriched with prebiotics or probiotics have some preventive benefits for atopic dermatitis; but, there is not enough evidence to recommend their routine use	220	7.70	11.4	80.9	4.1 (0.89)	3.7 (1.00)	4.1 (0.92)	*
For infants at high atopic risk fed exclusively with breast milk, the mother is advised to exclude cow’s milk and its derivatives from her diet in order to prevent primary sensitization to dairy proteins	218	54.60	17.4	28.0	2.5 (1.40)	3.0 (1.47)	2.0 (1.22)	*
If an infant cannot receive breast milk from birth, the most effective alternative to prevent sensitization to CMPA is to give him adapted cow’s milk formula continuously from birth	216	27.80	14.8	57.4	3.5 (1.44)	3.8 (1.32)	3.4 (1.52)	-
The use of rice/soy protein formulas is indicated for the primary prevention of CMPA in newborns and infants at atopic risk	218	67.90	17.0	15.2	2.1 (1.16)	2.2 (1.30)	2.0 (1.10)	-

**Table 4:** Results of the “Diagnosis” domain. Answers range from one (completely disagree) to five (completely agree). Means and SDs for each category. Overall data, categorized by work setting and significance for comparison ** *p* < 0.001.

Diagnosis	Overall					Primary Care	Hospital	
Item	N	Disagree (%)	Neutral (%)	Agree (%)	Mean (SD)	Mean (SD)	Mean (SD)	
Diagnosis of cow’s milk protein allergy is primarily based on the positivity of tests determining allergen-specific IgE	220	46.80	20.0	33.2	2.7 (1.46)	2.8 (1.45)	2.6 (1.44)	-
Symptom improvement upon milk withdrawal is sufficient most of the time to confirm CMPA diagnosis	222	25.70	22.5	51.8	3.3 (1.28)	3.9 (0.98)	3.0 (1.40)	**
For considering the effectiveness of cow’s milk withdrawal from the diet, it must be maintained for at least one month	222	20.30	16.2	63.5	3.6 (1.32)	4.0 (1.26)	3.5 (1.38)	**
In infants, the rapid onset of symptoms (immediate or up to 2 h) after the intake of common infant formula or the intake of foods containing cow’s milk is strongly suggestive of an IgE-mediated CMPA diagnosis	222	3.60	1.8	94.6	4.6 (0.74)	4.8 (0.41)	4.6 (0.81)	-
Systematic rejection of feeding bottles, accompanied by crying and irritability, with no other signs of illness, is symptomatic of CMPA	222	9.00	27.0	63.9	3.7 (0.89)	4.1 (0.79)	3.6 (0.93)	**
SPT with cow’s milk and CMP (alpha-lactalbumin, beta-lactoglobulin, and casein) is a valid diagnostic method for infants at any age	220	19.10	20.0	60.9	3.7 (1.15)	3.2 (1.24)	3.8 (1.12)	**
Disappearance of symptoms or clear clinical improvement in infants after replacing cow’s milk formula with an eHF can confirm the diagnosis of CMPA	222	9.00	12.2	78.8	4.1 (0.98)	4.2 (0.76)	3.9 (1.15)	-

**Table 5:** Results of the “Nutritional Management” domain. Answers range from one (completely disagree) to five (completely agree). Means and SDs for each category. Overall data, categorized by work setting and significance for comparison * *p* < 0.05; ** *p* < 0.001.

Nutritional Management	Overall					Primary Care	Hospital	
Variable	N	Disagree (%)	Neutral (%)	Agree (%)	Mean (SD)	Mean (SD)	Mean (SD)	
Current nutritional management of CMPA is based on the strict removal of milk proteins from the diet and education about foods that may contain them or other cross-reactive proteins	222	1.80	1.8	96.4	4.7 (0.58)	4.8 (0.40)	4.7 (0.74)	-
Oral desensitization protocols are effective in a significant percentage of cases, regardless of the pathogenic mechanism causing the allergy	222	17.10	32.9	50.0	3.4 (1.12)	3.4 (1.11)	3.3 (1.19)	-
IgE-mediated forms generally develop tolerance before non-IgE-mediated forms	222	58.50	19.4	22.1	2.3 (1.25)	2.3 (1.24)	2.1 (1.24)	-
At 2 years of life, 80% of infants with CMPA have developed tolerance to cow’s milk	222	5.90	7.7	86.5	4.3 (0.86)	4.4 (0.83)	4.5 (0.61)	-
Infants who develop CMPA while exclusively breastfeeding have a greater risk of developing late tolerance (>12 months) than those who develop the allergy when given formula	220	36.80	21.8	41.3	3.0 (1.23)	3.3 (1.06)	2.9 (1.31)	*
Cow’s milk should never be eliminated from the diet based on the presence of IgE sensitization (SPT or specific IgE test in serum) if the patient is consuming it with good tolerance.	220	12.70	15.0	72.3	3.9 (1.19)	3.6 (1.27)	4.3 (1.02)	**
In cases where the patient is consuming milk proteins with good tolerance, an elimination diet could lead to a loss of tolerance and the onset of an adverse reaction	218	16.50	18.3	65.1	3.8 (1.26)	3.7 (1.20)	4.0 (1.21)	*
Extensively hydrolyzed formulas (eHFs) are the first choice alternative in the nutritional management of cow’s milk allergy, especially in infants and young children	220	0.90	4.5	94.6	4.7 (0.65)	4.7 (0.79)	4.7 (0.57)	-
CMPA persistence is not related to the patient’s casein-specific IgE levels	214	46.70	22.9	30.4	2.7 (1.22)	3.1 (1.24)	2.2 (1.12)	**
In infants under 6 months of age, the use of hydrolyzed rice protein formulas is preferred over soy protein formulas.	220	4.10	2.7	93.1	4.5 (0.88)	4.5 (0.64)	4.5 (1.03)	-
Hydrolyzed rice protein formulas are organoleptically better accepted by infants than other extensively hydrolyzed formulas	220	6.80	10.0	83.2	4.2 (1.02)	4.2 (0.99)	4.1 (1.13)	-
Approximately 25% of infants require nutritional management with an elemental formula because they do not tolerate extensively hydrolyzed formulas	218	33.10	21.1	45.9	3.2 (1.26)	3.3 (1.34)	3.0 (1.29)	-
Extensively hydrolyzed formulas containing lactose are safe in children with an anaphylactic sensitization to cow’s milk proteins	214	22.00	23.8	54.2	3.5 (1.24)	3.5 (1.36)	3.5 (1.28)	-
Soy-based formulas are not considered nutritionally appropriate for infants under 6 months of age due to their phytoestrogen content	218	9.20	12.4	78.4	4.2 (1.11)	4.2 (1.05)	4.3 (1.10)	-
There is no evidence to demonstrate that certain probiotics promote the accelerated development of tolerance in infants with CMPA	218	36.70	27.1	36.2	3.1 (1.27)	3.2 (1.34)	2.9 (1.27)	-

**Table 6:** Results of the “Challenge” domain. Answers range from one (completely disagree) to five (completely agree). Means and SDs for each category. Overall data, categorized by work setting and significance for comparison * *p* < 0.05; ** *p* < 0.001.

Challenge	Overall					Primary Care	Hospital	
Variable	N	Disagree	Neutral	Agree	Mean (SD)	Mean (SD)	Mean (SD)	
Performing a challenge test is essential for the clear diagnosis of CMPA	220	22.80	16.4	60.9	3.6 (1.40)	3.5 (1.46)	3.8 (1.36)	-
Challenge tests cannot be overlooked under any circumstance	218	53.70	22.0	24.3	2.4 (1.36)	2.6 (1.42)	2.0 (1.20)	*
After a positive diagnostic challenge test, an exclusion diet should be maintained for at least 6 months	220	12.20	5.5	82.2	4.1 (1.08)	4.2 (1.04)	4.1 (1.00)	-
After a positive diagnostic challenge test, no further challenge should be performed before 12 months of age	218	28.90	13.3	57.8	3.4 (1.37)	3.9 (1.24)	3.1 (1.42)	**
Challenge tests should always be performed in a hospital setting	218	26.10	12.4	61.5	3.6 (1.53)	3.7 (1.35)	3.5 (1.65)	-
Negative levels in the SPT and serum IgE test are not necessary before tolerance is verified by a controlled challenge test	217	29.50	18.4	52.1	3.3 (1.29)	3.7 (1.07)	3.2 (1.42)	*
If the controlled oral food challenge test is negative, followed by regular intake of cow’s milk with good tolerance at home for two weeks, even if IgE sensitization in the SPT or serum IgE persists, it can be considered that cow’s milk allergy is in clinical remission and it is allowed to be incorporated it into the diet	219	0.90	6.4	92.7	4.3 (0.68)	4.3 (0.68)	4.5 (0.54)	-
A cautious and controlled oral food challenge test for milk is the only definitive tool to assess whether progression to tolerance has occurred	220	4.50	6.8	88.6	4.4 (0.81)	4.4 (0.66)	4.6 (0.60)	*

## Data Availability

The raw data supporting the conclusions of this article will be made available by the authors, without undue reservation.

## Supplementary Materials

The following supporting information can be downloaded at: https://www.mdpi.com/article/10.3390/nu15163586/s1, File S1: Final questionnaire for the project. References [36,37,38,39,40,41] are cited in the Supplementary Materials.
