# Supplementary material for: Spanish Pediatricians’ Positions Regarding Prevention, Diagnosis, Nutritional Management, and Challenges in Cow’s Milk Protein Allergy
Source: Nutrients. 2023 Aug 15;15(16):3586. doi: 10.3390/nu15163586 (PMC10458855; doi:10.3390/nu15163586)
Supplement: Supplementary file 1 [file nutrients-15-03586-s001.zip › nutrients-2472982-supplementary.pdf]

Estudio PHASE

Perfil de uso de fórmulas a base de
Hidrolizados extensos,
Arroz,
Soja y
Elementales en la APLV

Introducción

La alergia a la proteína de la leche de vaca es la alergia más común en bebés y niños pequeños[1-2]. La reacción inmunitaria puede ser mediada por IgE, no mediada por IgE, o mixta lo que comportará diferencias en su sintomatología y gravedad de los síntomas. Aunque es una entidad prevalente, su manejo varía de manera significativa dependiendo de la experiencia y la especialidad del clínico a cargo del paciente. Recientemente, distintas organizaciones han publicado varias guías en revistas de alto impacto en un intento de unificar el manejo de esta patología por los especialistas pediátricos implicados, incluyendo pediatras de atención primaria, gastroenterólogos y alergólogos pediátricos[3-8].

En este contexto proponemos realizar un estudio para determinar opiniones y actitudes del pediatra sobre la APLV y su manejo en la consulta y compararlas con las registradas por otros especialistas[9-10].

Del mismo modo existe cierta discusión con el momento de utilización de las diferentes alternativas terapéuticas, como son los hidrolizados extensos a partir de proteínas de leche de vaca, los hidrolizados vegetales de arroz y de soja o las fórmulas elementales en nuestro entorno.

Objetivo general

- Analizar las intervenciones terapéuticas en los niños con APLV en las consultas de pediatría y en particular del uso de hidrolizados vegetales.

Objetivos específicos

- Valoración del perfil de actitud y percepción actual de los pediatras españoles respecto a la APLV en comparación a otras encuestas previas realizadas en especialistas[1-2].

Metodología

La población de estudio son pediatras que tratan a niños con APLV. No se incluyen reacciones adversas a leche de vaca sin mecanismo inmunológico, como la intolerancia a la lactosa u otras.

Se precisa que el pediatra participante tenga experiencia en el manejo de la APLV y haya utilizado en algún caso un hidrolizado vegetal para analizar diferencias en su uso respecto a otras fórmulas.

Diseño del estudio

- Estudio descriptivo, transversal y multicéntrico realizado en consultas de pediatría de centros de salud en España.
- El pediatra participante cumplimentará una encuesta sobre hábitos y actitud general frente a la APLV.

Referencias

1. Sanz J, Martorell A, Michavila A, Nieto A. Incidence of IgE- mediated allergy to cow's milk proteins in the first year of life. An Esp Pediatr. 2001; 54:536-9.
2. Garcia Ara MC, Boyado MT, Diaz Pena JM, Martin Muñoz F, Pascual C, Garcia Sanchez G, et al. Incidencia de alergia a leche de vaca y su repercusión en el consumo de hidrolizados. An Pediatr. 2003; 58:100-5.
3. Fiocchi A, Brozek J, Schünemann H, Bahna SL, von Berg A, Beyer K, et al. World Allergy Organization (WAO) diagnosis and ratio- nale for action against cow's milk allergy (DRACMA) guidelines. World Allergy Organ J. 2010; 21:1-125.
4. Vandenplas Y, Brueton M, Dupont C, Hill D, Isolauri E, Koletzko S, et al. Guidelines for the diagnosis and management of cow's milk protein allergy in infants. Arch Dis Child. 2007; 92:902-8.
5. Montijo-Barrios E, López-Ugalde MV, Ramírez-Mayans J, Anaya- Flórez MS, Arredondo-García JL, Azevedo-Tenorio I, et al. Guía latinoamericana para el diagnóstico y tratamiento de alergia a las proteínas de la leche de vaca (GL-APLV). Rev Invest Clin. 2014; 66:9-72.
6. Caffarelli C, Baldi F, Bendandi B, Calzone L, Marani M, Pasqui- nelli P. Cow's milk protein allergy in children: a practical guide. Ital J Pediatr. 2010; 36:5.
7. Martorell-Aragonés A, Echevarría-Zudaire L, Alonso-Lebrero E, Boné-Calvo J, Martín-Muñoz MD, Nevot-Falcó S, et al. Position document: IgE-mediated cow's milk allergy. Food allergy committee of SEICAP (Spanish Society of Pediatric Allergy, Asthma and Clinical Immunology). Allergol Immunopathol. 2015; 43:507-26.
8. Espín Jaime, B., Díaz Martín, J. J., Blesa Baviera, L. C., Claver Monzón, Á et al. Non-IgE-mediated cow's milk allergy: Consensus document of the Spanish Society of Paediatric Gastroenterology, Hepatology, and Nutrition (SEGHNP), the Spanish Association of Paediatric Primary Care (AEPAP), the Spanish Society of Extra-hospital Paediatric. Anales de Pediatria 2019, 90(3), 193.e1-193.e11.
9. Alonso-Lebrero, E., Bento, L., Martorell-Aragonés, A., & Ribeiro, L. Iberian consensus on cow's milk allergy: The CIBAL Study. Allergologia et Immunopathologia 2018, 46(6), 517-532.
10. Pascual Pérez, A. I., Méndez Sánchez, A., Segarra Cantón, Ó., Espin Jaime, B., Jiménez Treviño, S., Bousoño García, C., & Díaz Martín, J. J. Attitudes towards cow's milk protein allergy management by spanish gastroenterologist. Anales de Pediatria 2018, 89(4), 222-229.

Variabilidad en la actitud y conocimientos en la alergia a la proteína de leche de vaca

Cuestionario de opinión y práctica clínica en la APLV

El siguiente cuestionario se procesará de forma totalmente anónima. Está compuesto por preguntas de respuesta abierta y respuestas de escala. Su cumplimentación requiere de un tiempo aproximado de 15 minutos. Gracias por su participación.

Datos de filiación

1. **Género** ☐ **Femenino** ☐ **Masculino**

2. **Edad_______años**

3. **Ámbito laboral** ☐ **Público** ☐ **Privado** ☐ **Ambos**

4. **Centro en donde ejerce** ☐ **Atención primaria** ☐ **Hospitalario** ☐ **Ambos**

5. **Zona de referencia**
   ☐ Medio rural (municipio de < 2.000 habitantes)
   ☐ Medio semiurbano (municipio entre 2.000 y 10.000 habitantes)
   ☐ Medio urbano: (municipio con más de 10.000 habitantes)

6. **Indicar especialidad** ___________________________________

7. **Años de experiencia** (incluidos los años de formación MIR)_______**años**

8. **Ante un cuadro clínico sugerente de alergia mediada por IgE ¿qué prueba complementaria solicita:**
   ☐ Prick test a PLV ☐ IgE específica a PLV en sangre ☐ Ambas

9. **¿Se realizan en su centro pruebas epicutáneas (patch test)?**
   ☐ No ☐ Sí

10. **¿Quién realiza la prueba de provocación oral en su Centro?**
   ☐ Alergia ☐ Gastro
   ☐ Pediatría hospitalaria ☐ Pediatría de atención primaria
   ☐ En mi centro no se realizan

11. En general, **¿Qué tipo de fórmula recomienda inicialmente en menores de 6 meses con alergia IgE mediada?**

- ☐ Fórmulas extensamente hidrolizadas/semielementales
- ☐ Fórmulas vegetales con proteínas hidrolizadas de arroz
- ☐ Fórmulas vegetales con proteínas de soja (intactas o hidrolizadas)
- ☐ Fórmulas elementales
- ☐ Otras: ______________________________

12. En general, **¿Qué tipo de fórmula recomienda inicialmente en menores de 6 meses con alergia NO IgE mediada?**

- ☐ Fórmulas extensamente hidrolizadas/semielementales
- ☐ Fórmulas vegetales con proteínas hidrolizadas de arroz
- ☐ Fórmulas vegetales con proteínas de soja (intactas o hidrolizadas)
- ☐ Fórmulas elementales
- ☐ Otras: ______________________________

13. En general, **¿Qué tipo de fórmula recomienda inicialmente en mayores de 6 meses con alergia IgE mediada?**

- ☐ Fórmulas extensamente hidrolizadas/semielementales
- ☐ Fórmulas vegetales con proteínas hidrolizadas de arroz
- ☐ Fórmulas vegetales con proteínas de soja (intactas o hidrolizadas)
- ☐ Fórmulas elementales
- ☐ Otras: ______________________________

14. En general, **¿Qué tipo de fórmula recomienda inicialmente en mayores de 6 meses con alergia NO IgE mediada?**

- ☐ Fórmulas extensamente hidrolizadas/semielementales
- ☐ Fórmulas vegetales con proteínas hidrolizadas de arroz
- ☐ Fórmulas vegetales con proteínas de soja (intactas o hidrolizadas)
- ☐ Fórmulas elementales
- ☐ Otras: ______________________________

15. **Señale el grado de acuerdo que tiene con las siguientes afirmaciones**
(desde 0= nada de acuerdo a 5= totalmente de acuerdo).

1. Usted cree que sus conocimientos sobre APLV son adecuados.

☐1 ☐2 ☐3 ☐4 ☐5

Diagnóstico

2. El diagnóstico de la alergia a proteínas de leche de vaca se basa fundamentalmente en la positividad de las pruebas que determinan IgE específica.

☐1 ☐2 ☐3 ☐4 ☐5

3. La mejoría de los síntomas al retirar la leche es suficiente la mayoría de las veces para confirmar el diagnóstico de APLV.

1 2 3 4 5

4. Para considerar que la eliminación de la leche de vaca de la dieta es eficaz, debe mantenerse al menos un mes.

1 2 3 4 5

5. En los bebés, la rápida aparición de los síntomas (inmediata o hasta 2 horas) después de tomar una fórmula láctea o ingerir alimentos que contienen leche de vaca es muy sugestiva de un diagnóstico de APLV mediada por IgE.

1 2 3 4 5

6. El rechazo sistemático del biberón, acompañado de llanto e irritabilidad, sin otros signos de enfermedad, son sintomáticos de APLV.

1 2 3 4 5

7. La prueba de punción cutánea (Skin Prick Test) con leche de vaca y CMP (alfa-lactoalbúmina, beta-lactoglobulina y caseína) son un método de diagnóstico válido para bebés de cualquier edad.

1 2 3 4 5

8. La desaparición de los síntomas o una clara mejoría clínica en los bebés después de reemplazar una fórmula de leche de vaca adaptada por un eHF puede confirmar el diagnóstico de APLV.

1 2 3 4 5

Tratamiento

9. El tratamiento actual de APLV se basa en la eliminación estricta de las proteínas lácteas de la dieta, la educación sobre alimentos que pueden contener estas u otras proteínas con una reacción cruzada, lo que indica una alternativa apropiada para garantizar una nutrición adecuada, el seguimiento del crecimiento, el entrenamiento para detectar síntomas de ingestión accidental y el tratamiento adaptado a la gravedad de los síntomas.

1 2 3 4 5

10. Los protocolos de desensibilización oral son eficaces en un porcentaje significativo de casos independientemente del mecanismo patogénico que produzca la alergia.

1 2 3 4 5

11. Las formas mediadas por IgE por lo general desarrollan tolerancia antes que las no mediadas por IgE.

1 2 3 4 5

12. A los 2 años de vida el 80% de los lactantes con APLV han desarrollado tolerancia a la leche de vaca.

1 2 3 4 5

13. Los lactantes que desarrollan APLV mientras reciben LM exclusiva, tienen un mayor riesgo de desarrollar tolerancia tardía (> 12 meses) que aquellos que desarrollan la alergia al recibir fórmulas para lactantes.

1 2 3 4 5

14. La leche de vaca nunca debe eliminarse de la dieta en función de la existencia de sensibilización IgE (SKin Prick Test o IgE específico del suero) si el paciente la está consumiendo con buena tolerancia.

1 2 3 4 5

15. En los casos en que el paciente está consumiendo proteínas lácteas con buena tolerancia, la dieta de eliminación podría conducir a una pérdida de tolerancia y la aparición de una reacción adversa potencialmente grave cuando se reintroduce en la dieta.

1 2 3 4 5

16. Los hidrolizados extensos (eHF) son la primera opción como alternativa en el tratamiento de la alergia a la leche de vaca, especialmente en lactantes y niños pequeños.

1 2 3 4 5

17. La persistencia de la APLV no se relaciona con los niveles de IgE específica a caseína del paciente.

1 2 3 4 5

18. En niños menores de 6 meses la utilización de fórmulas de arroz hidrolizado está indicada por encima de las de soja.

1 2 3 4 5

19. Las fórmulas de arroz hidrolizado son organolépticamente mejor aceptadas por el niño que otras fórmulas extensamente hidrolizadas.

1 2 3 4 5

20. El 25% de los lactantes precisan tratamiento con una fórmula elemental por no tolerar los hidrolizados de alto grado.

1 2 3 4 5

21. Las fórmulas a base de hidrolizados extensos con lactosa son seguras en niños con sensibilización anafiláctica a las proteínas de la leche de vaca.

1 2 3 4 5

22. Las fórmulas a base de soja no se consideran nutricionalmente adecuadas para lactantes menores de 6 meses de edad debido a su contenido de fitoestrógenos y fitoestrógenos.

1 2 3 4 5

23. No hay evidencias que demuestren que determinados probióticos favorezcan el desarrollo acelerado de tolerancia en los lactantes con APLV.

1 2 3 4 5

Prevención

24. Las fórmulas parcialmente hidrolizadas y extensamente hidrolizadas tienen un efecto preventivo de la enfermedad atópica en comparación con las fórmulas que contienen proteínas intactas.

1 2 3 4 5

25. Las fórmulas de leche hidrolizadas extensamente administradas a lactantes con alto riesgo atópico desde el nacimiento hasta 6 meses para complementar o reemplazar la leche materna son más eficaces que las fórmulas parcialmente hidrolizadas para prevenir la APLV.

1 2 3 4 5

26. La fórmula enriquecida con prebióticos o probióticos tiene algunos beneficios preventivos para la dermatitis atópica, pero no hay suficiente evidencia para recomendar su uso rutinario.

1 2 3 4 5

27. Para los lactantes con alto riesgo atópico alimentados exclusivamente con leche materna, se recomienda excluir la leche de vaca y sus derivados de la dieta materna para prevenir la sensibilización primaria de proteínas lácteas.

1 2 3 4 5

28. Si un bebé no puede recibir leche materna desde el nacimiento, la alternativa más eficaz para evitar la sensibilización a APLV es administrar una fórmula de leche de vaca adaptada continuamente desde el nacimiento.

1 2 3 4 5

29. El uso de fórmulas a base de arroz/soja está indicado para la prevención primaria de APLV en recién nacidos y lactantes con riesgo atópico.

1 2 3 4 5

Provocación

30. La realización de una prueba de provocación es imprescindible para el diagnóstico de certeza de APLV.

1 2 3 4 5

31. La prueba de provocación no puede ser obviada en ninguna circunstancia.

1 2 3 4 5

32. Tras una prueba de provocación diagnóstica positiva, se debe mantener una dieta de exclusión durante al menos 6 meses.

1 2 3 4 5

33. Tras una prueba de provocación diagnóstica positiva, no se debe realizar otra prueba de provocación antes de los 12 meses de vida.

1 2 3 4 5

34. La prueba de provocación debe realizarse siempre en medio hospitalario.

1 2 3 4 5

35. No es necesario esperar niveles negativos de Skin Prick Test y IgE sérico antes de comprobar la tolerancia mediante una prueba de desafío controlada.

1 2 3 4 5

36. Una prueba de provocación oral controlada negativa, seguida de la ingestión regular de leche de vaca con buena tolerancia en casa durante dos semanas, nos permite considerar la alergia a la leche de vaca como en remisión clínica y permite que se incorpore a la dieta, incluso si persiste la sensibilización IgE en Skin Prick Test o IgE sérica.

1 2 3 4 5

37. Una prueba de provocación oral cautelosa y controlada para la leche es la única herramienta definitiva para evaluar si se ha producido una progresión hacia la tolerancia.

1 2 3 4 5

Muchas gracias por su participación.
